# LncRNA NR_045147 modulates osteogenic differentiation and migration in PDLSCs via ITGB3BP degradation and mitochondrial dysfunction

**DOI:** 10.1093/stcltm/szae088

**Published:** 2024-12-14

**Authors:** Lujue Long, Chen Zhang, Zhengquan He, Ousheng Liu, Haoqing Yang, Zhipeng Fan

**Affiliations:** Laboratory of Molecular Signaling and Stem Cells Therapy, Beijing Key Laboratory of Tooth Regeneration and Function Reconstruction, Capital Medical University School of Stomatology, Beijing, People’s Republic of China; Laboratory of Molecular Signaling and Stem Cells Therapy, Beijing Key Laboratory of Tooth Regeneration and Function Reconstruction, Capital Medical University School of Stomatology, Beijing, People’s Republic of China; Department of Orthodontics, Changsha Stomatology Hospital, Changsha, Hunan, People’s Republic of China; Hunan Key Laboratory of Oral Health Research, Hunan 3D Printing Engineering Research Center of Oral Care, Academician Workstation for Oral-Maxilofacial and Regenerative Medicine, Hunan Clinical Research Center of Oral Major Diseases and Oral Health, Xiangya Stomatological Hospital, Xiangya School of Stomatology, Central South University, Changsha, Hunan, People’s Republic of China; Laboratory of Molecular Signaling and Stem Cells Therapy, Beijing Key Laboratory of Tooth Regeneration and Function Reconstruction, Capital Medical University School of Stomatology, Beijing, People’s Republic of China; Laboratory of Molecular Signaling and Stem Cells Therapy, Beijing Key Laboratory of Tooth Regeneration and Function Reconstruction, Capital Medical University School of Stomatology, Beijing, People’s Republic of China; Beijing Laboratory of Oral Health, Capital Medical University, Beijing, People’s Republic of China; Research Unit of Tooth Development and Regeneration, Chinese Academy of Medical Sciences, Beijing, People’s Republic of China

**Keywords:** cell migration, gene therapy, tissue regeneration, animal models, differentiation

## Abstract

Periodontitis is an inflammation of the alveolar bone and soft tissue surrounding the teeth. Although mesenchymal stem cells (MSCs) have been implicated in periodontal regeneration, the mechanisms by which they promote osteogenesis remain unclear. We examined whether epigenetic modifications mediated by the long-noncoding RNA (lncRNA) NR_045147, which plays a crucial role in cancer, influence the osteogenic differentiation of periodontal ligament stem cells (PDLSCs). Alkaline phosphatase staining, alizarin red staining, and western blotting were used to detect the effects of NR_045147 on PDLSC osteogenic differentiation. Scratch migration and transwell chemotaxis assays were used to evaluate the effects of NR_045147 on PDLSC migration. Mitochondrial function was evaluated via Seahorse XF analysis to measure changes in cellular respiration upon manipulation of NR_045147 expression. Ubiquitination assays were performed to examine the protein stability and degradation pathways affected by the NR_045147–MDM2 interaction. An in vivo nude rat calvarial defect model was established and gene-edited PDLSCs were re-implanted to examine the osteogenic effects of NR_045147. NR_045147 significantly reduced PDLSC osteogenic differentiation and migration ability both in vitro and in vivo. Under inflammatory conditions, the loss of NR_045147 rescued osteogenesis. NR_045147 significantly blocked the expression of integrin beta3-binding protein (ITGB3BP). Mechanistically, NR_045147 promoted the ITGB3BP-MDM2 interaction, thus increasing ITGB3BP ubiquitination and degradation. NR_045147 regulated PDLSC mitochondrial respiration and ITGB3BP upregulation efficiently promoted their osteogenic differentiation and migration ability. Concluding, NR_045147 downregulation enhances PDLSC osteogenic differentiation and migration, connects changes in cellular metabolism to functional outcomes via mitochondrial respiration, and promotes ITGB3BP degradation by mediating its interaction with MDM2.

## Graphical Abstract

**Graphical Abstract:**
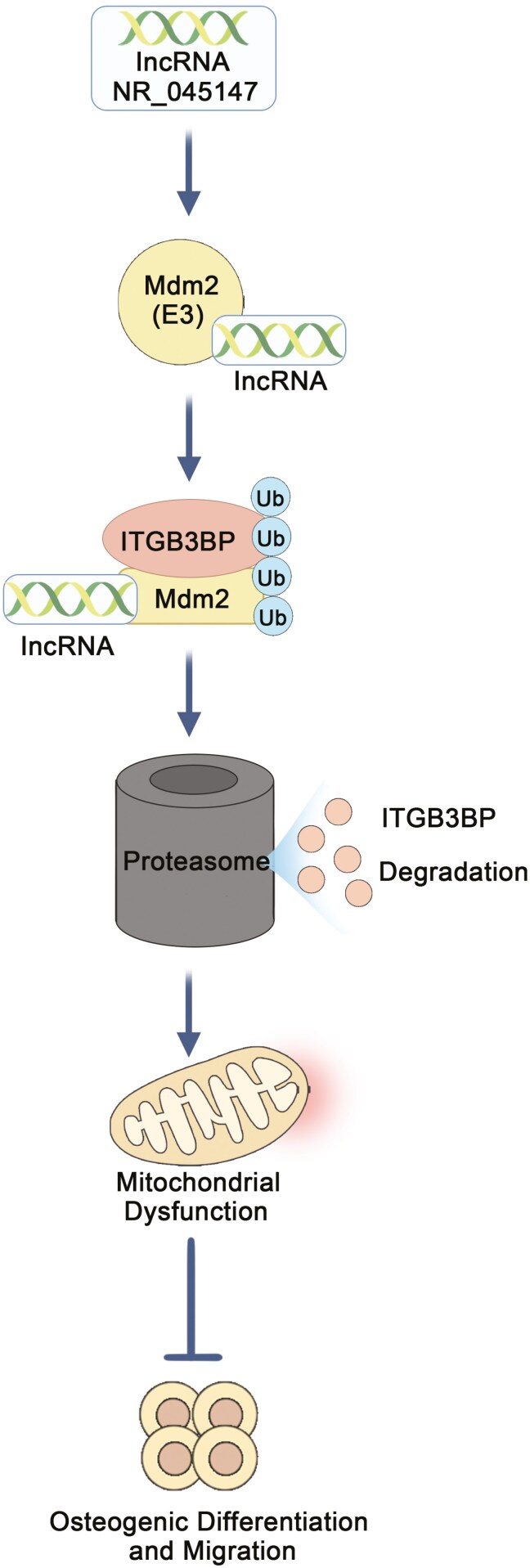

**Significance statement**

Although mesenchymal stem cells have been implicated in periodontal regeneration, the mechanisms whereby they promote osteogenesis remain unclear. These findings highlight a potential stem cell therapeutic target for periodontitis.

## Introduction

Periodontitis, an inflammatory disease with a high prevalence and the most common cause of tooth loss, afflicts both younger and older populations in both developed and developing countries.^1,2^ Periodontitis leads to the loss of attachment of the periodontium, progressing to alveolar bone loss and potentially resulting in the loss of the affected tooth.^3^ Severe periodontitis is associated with other chronic inflammatory diseases, including low oxygen saturation, adverse pregnancy, cardiovascular and respiratory diseases, Alzheimer’s disease, and cancer.^4,5^ Treating periodontitis could thus reduce the risk of related local and systemic comorbidities. However, the current treatments for periodontitis, which include surgical intervention, physiotherapy, and pharmacological approaches,^6^ are not sufficiently effective.^7^ Mesenchymal stem cell (MSC)-based therapy has generated promising prospects for enhancing the efficacy of periodontal bone-ligament-cementum regeneration in both hard and soft tissues.^8^

Although the clinical transfer of MSCs has demonstrated positive effects in treating chronic inflammatory diseases, concerns remain regarding the difficulties in promoting MSC differentiation, along with the potential for immune rejection.^9^ Periodontal ligament MSCs (PDLSCs) are particularly important for the treatment of periodontitis because of their unique localization and potential for regenerative therapy. They can differentiate into osteoblasts, cementoblasts, and fibroblasts, which are crucial for periodontal tissue repair and regeneration. By harnessing their therapeutic potential, innovative strategies can be developed to effectively address periodontitis, potentially leading to improved clinical outcomes and enhanced tissue regeneration.^10^ Gene therapy, based on stem cell therapy, can precisely regulate the microenvironment of the defect to enhance periodontal regeneration.^11^ For periodontal repair, the gene can be injected directly into the periodontal defect via a retrovirus, or first incorporated into stem cells. These cells are then proliferated and delivered directly into the defect. Thus, identifying key targets and regulatory mechanisms underlying PDLSC osteogenic differentiation may improve bone regeneration in periodontitis.

Recently, several long-noncoding RNAs (lncRNAs) have been identified, characterized by lengths exceeding 200 nucleotides and an absence of protein-coding potential.^12^ Different patterns of lncRNA expression regulate the cell cycle, proliferation, metastasis, immunobiology, and differentiation.^13^ CYTOR, a lncRNA, positively modulates SOX11 expression by competitively binding to miR-6512-3p, thereby promoting PDLSC osteogenic differentiation.^14^ Downregulation of SNHG8, a lncRNA, reduces the expression of the enhancer of zeste homolog 2 (EZH2), thereby negatively regulating PDLSC osteogenic differentiation.^15^ By binding directly to PKM1/2 proteins, the lncRNA GACAT2 reverses inflammation-induced damage to mitochondrial function in PDLSCs and cementoblastic differentiation.^16^ The overexpression of LncRNA LOXL1-AS1 increases the Bax/Bcl-2 ratio and caspase-3 levels, potentially ameliorating periodontitis by downregulating interleukin (IL)-1β.^17^

We have previously comprehensively analyzed lncRNA differential expression in regions associated with osteogenic development in bone marrow MSCs (BMSCs). Among these lncRNAs, NR_045147, which is 1011 nt long and located on chromosome 1, exhibited significantly higher expression in PDLSCs than in BMSCs.^18^ NR_045147 is a transcript variant of the integrin subunit beta 3 binding protein (ITGB3BP), which possesses an additional exon. This variant is considered noncoding because its use of the 5ʹ-most expected translational start codon renders it a candidate for nonsense-mediated mRNA decay. ITGB3BP may be involved in mitotic progression.^19^ As a prognostic hub gene, its expression is upregulated in hepatocellular carcinoma tissue.^20^ It interacts with centrosomal ninein protein, contributes to human umbilical vein endothelial cell proliferation and formation of vascular structures,^21^ and participates in cell adhesion and signaling, which are crucial in the pathogenesis of periodontitis and osteogenic differentiation of PDLSC. NR_045147 expression was reduced upon osteogenic induction,^22^ suggesting its potential regulatory function in osteogenic differentiation of PDLSC. This finding prompted us to examine its role in periodontitis and its potential as a therapeutic target for promoting periodontal tissue regeneration. Cellular ITGB3BP levels were altered by modulating NR_045147 expression. Our objective was to examine the effects of the NR_045147 on PDLSC osteogenic differentiation and migration. This study aimed to enhance our understanding of MSC regulation and bone regeneration by elucidating the regulatory functions of the NR_045147.

## Materials and methods

### Cell cultures

We obtained PDLSCs in accordance with the guidelines of the International Society for Stem Cell Research (ISSCR), with patient consent. The study was approved by the ethics committee of Beijing Stomatological Hospital, Capital Medical University, China. We selected patients who were healthy, without major communicable diseases. The extracted teeth were washed with phosphate-buffered saline (PBS) supplemented with 1% penicillin-streptomycin. The periodontal ligaments were rapidly peeled from the middle third of the root and digested in 3 mg/mL of type I collagenase (Sigma-Aldrich) and 4 mg/mL of dispase (Roche Diagnostics GmbH) at 37 °C for 40 min. Single-cell suspensions were successfully obtained using a 70-mm strainer (Nest Biotechnology). The cells were grown in an α-MEM medium (Gibco, Thermo Fisher Scientific) supplemented with 10 000 units/mL of penicillin-streptomycin (Gibco), 200 mM l-glutamine (Gibco) and 10% fetal bovine serum (Gibco) in an incubator with 5% CO_2_ at 37 °C. The cells collected between passages 3 and 5 were used for subsequent experiments.

### Plasmid construction and viral infection

Recombinant plasmids of NR_045147 were created following standard protocols and verified by gene sequencing. Lentiviruses carrying shRNA or overexpressing the NR_045147 vector were purchased from Shanghai GeneChem. NR_045147 cDNA was subcloned into the GV367 lentiviral vector to obtain LV-NR_045147 for overexpression. A lentiviral blank vector was used as a negative control (LV-vector). To examine the effects of NR_045147 on PDLSCs in vitro, we knocked down NR_045147 by transfecting lentivirus plasmid containing short hairpin RNA targeting NR_045147 (LV3-NR_045147sh) into PDLSCs. Cells transfected with blank plasmid were used as negative controls (LV3-Consh). PDLSCs were seeded and cultured in 100-mm dishes with 8 μg/mL polybrene, infected with lentiviruses, and incubated overnight. The transfected PDLSCs were then selected using the appropriate antibiotics after 72 hours of incubation with the lentivirus. The shRNA target sequences were as follows: control shRNA (Consh), 5ʹ-TTCTCCGAACGTGTCACGTTTC-3ʹ, and NR_045147 shRNA (NR_045147sh), 5ʹ- GCCTCAGATGTCACAACCTCT-3ʹ.

### Alkaline phosphatase (ALP) quantification and alizarin red staining

Periodontal ligament stem cells were stimulated for 3 days, and ALP activity was recorded at 405 nm using an ALP activity assay kit (Sigma-Aldrich). ALP activity was normalized to the total protein content. Total protein levels were quantified using the BCA Protein Quantitation Assay kit (KeyGen). For the induction of osteogenic differentiation, 100% confluent BMSCs were maintained in an osteoblastic medium (OM) containing 10% FBS, 0.2 mM ascorbic acid, 10 mM β-glycerophosphate, and 100 nM dexamethasone. Cells were cultured in OM for 14 days and stained with alizarin red. Transfected PDLSCs were soaked in ice-cold 70% ethyl alcohol for 1 hour and slowly rocked with 40 mM/L alizarin red (PH4.2; Sigma-Aldrich) for 10 minutes at room temperature. Next, alizarin red was dissolved in 10 mmol/L cetylpyridinium chloride (Sigma-Aldrich) for 30 minutes at 25°C. Optical density (absorbance) was measured at 562 nm. Calcium levels were normalised to those of the total protein content.

### Real-time reverse transcriptase-polymerase chain reaction

Total RNA was extracted using the TRIzol reagent (Invitrogen, Thermo Fisher Scientific). The mRNA (1 μg) was reverse transcribed into cDNA using the Prime Script RT Master Mix Kit (Vazyme). Real-time reverse transcriptase-polymerase chain reaction was performed using a SYBR Green PCR Kit (Qiagen) and an iCycler iQ Multi-color Real-Time PCR Detection System (Bio-Rad). Gene expression was calculated using the 2^−^^△△^^CT^ method and normalized to that of GAPDH. The primers used are listed in Table 1.

**Table 1.** Primers sequences used in the real-time RT-PCR.

Gene symbol	Primer sequences (5ʹ-3ʹ)
*GAPDH*-F	CGGACCAATACGACCAAATCCG
*GAPDH-R*	AGCCACATCGCTCAGACACC
*IL-6-F*	TGGTGTTGCCTGCTGCCTTC
*IL-6-R*	GCTGAGATGCCGTCGAGGATG
*IL-8-F*	ACCACACTGCGCCAACACAG
*IL-8-R*	AACTTCTCCACAACCCTCTGCAC
*LncRNA NR_045147-F*	CAGCCTCAGATGTCACAACC
*LncRNA NR_045147-R*	AAATGGCTTTAAGGAATTCATAGC
*ITGB3BP (NM-4288)-F*	AGTTGGATGGTCTGTTAGAAGA
*ITGB3BP (NM-4288) –R*	CAAGTTCCAGTTGTTGGAGAA

### Western blot analysis

Total cellular protein was harvested from PDLSCs using RIPA lysis buffer (Applygen, Beijing) and a protease inhibitor cocktail (MedChemExpress). Proteins (25 grams) were electrophoretically separated on a 10% sodium dodecyl sulphate-polyacrylamide gel electrophoresis (SDS-PAGE) gel and transferred to a polyvinylidene fluoride (PVDF) membrane using a semi-dry electrophoretic transfer apparatus (Bio-Rad). The membranes were blocked in 5% nonfat milk for 1 hour at room temperature, incubated overnight with primary antibodies at 4 °C, and then washed 4 times with Tris-buffered saline containing 0.05% Tween 20 (TBST) at 5 minutes intervals. Subsequently, they were incubated with secondary antibodies for 1 hour at 25 °C, followed by washing 4 times with TBST. Bands were visualized using enhanced chemiluminescence detection reagents (Applygen), with GAPDH as an internal control. Primary antibodies against the following proteins were used: bone sialoprotein (BSP; bs-2668R; Bioss), osteocalcin (OCN; bs-0470R; Bioss), osterix (OSX bs-1110R, Bioss), ITGB3BP (10743-1-AP; Proteintech), and actin (66009-1-Ig; Proteintech).

### Co-immunoprecipitation and ubiquitination-IP

The medium was replaced with a new medium containing MG132 (20 μM). The cells were incubated for 6 hours, rinsed with ice-cold PBS, and solubilized for 15 minutes on ice in cell lysis buffer for IP, followed by supplementation with protease inhibitors. MDM2, an important E3 ligase, affects ligase proliferation and may affect periodontal cell proliferation.^23^ The lysates were then centrifuged at 13 400 × *g* for 10 minutes, and the supernatants were immunoprecipitated at 4 °C with MDM2 (Co-IP) or ITGB3BP (Ubi-IP) antibodies (5 mg/mL) for 4 hours, followed by incubation overnight with protein A/G beads. After centrifugation, the beads were washed 5 times with RIPA buffer. Bound proteins were eluted with 2× SDS sample buffer and subjected to SDS-PAGE. Proteins resolved via SDS-PAGE were transferred to PVDF membranes. Blots were incubated at room temperature for 1 hour in 5% skim milk, followed by incubation with indicated antibodies at 4 °C overnight. The remaining steps were the same as those used for Western blotting.

### Mitochondrial respiration measurements

Mitochondrial oxygen consumption rate (OCR) was analyzed using a Seahorse XFe24 analyzer (Seahorse Bioscience). Periodontal ligament stem cells (6 × 10^4^) were placed into 24-well XF24 cell culture microplates (Seahorse Bioscience). After treatment, the medium was replaced with XF assay medium (Seahorse Bioscience) supplemented with 1 mM pyruvate, 2 mM glutamine, and 10 mM d-glucose. After placing the cell culture microplate into a non-CO_2_ incubator at 37 °C for 1 hour, OCR was measured using the Seahorse Bioscience XF24 Extracellular Flux Analyzer (Seahorse Bioscience). Measurements were obtained sequentially under 4 sets of conditions: (1) basal conditions, with no additives; (2) oligomycin (1 M) was added to reversibly inhibit adenosine triphosphate (ATP) synthase and OXPHOS to determine the effects of glycolysis alone; (3) carbonyl cyanide 4-(trifluoromethoxy)phenylhydrazone (2 μM), a mitochondrial uncoupler, was added to induce maximal respiration; and (4) rotenone/antimycin A (0.5 μM), a complex I inhibitor and mitochondrial poison, was added to end the reaction. Seahorse software was used to plot the results. OCR was normalized based on the cell protein concentration per well.

### Scratch migration assays

Periodontal ligament stem cells were seeded in 6-well culture plates with 2.5 × 10^5^ cells per well in a serum-free medium for 24 hours. Subsequently, the cells were scratched with a 1000-μL pipette tip (Axygen) to create a wound, washed twice with PBS to clear the floating cells, and then incubated in a fresh culture medium. Scratch images were observed under a microscope at 0 hour, 24 hours, and 48 hours. To analyze the results of snapshot pictures, the distance from one side of the wound to the other was measured using a scale bar. The relative wound width was calculated using the following formula: the original scratch width-the final scratch width. Wound closure was measured, and wound relative width was determined using the ImageJ Pro 1.49v software (National Institutes of Health).

### Transwell chemotaxis assays

Periodontal ligament stem cells were seeded in transwell chambers (with 8 -μm pores) with 2.5 × 10^4^ cells and cultured in 100 μL serum-free upper chambers. Subsequently, the bottom chambers were equipped with 600 μL of MEM Alpha medium containing 10% FBS. After 24 or 48 hours, the cells were fixed using 4% paraformaldehyde and stained with 0.5% crystal violet staining solution. Finally, PBS was used to clear the floating dye thrice, and 5 fields per chamber were counted using a microscope (Olympus, Tokyo, Japan) at 200× magnification.

### Immunofluorescence staining

Briefly, after antigen retrieval, the slices were blocked with 10% goat serum for 1 hour and then incubated with primary antibodies against OSX (ab209484, abcam) and BSP (A16220, abclonal) at 4 °C overnight. The next day, the slices were washed with PBST and incubated with an anti-rabbit fluorescein-conjugated antibody at room temperature for 1 hour, followed by nuclear staining with DAPI. A fluorescence microscope was used to capture the images.

### RNA pull-down assays

NR_045147 or antisense NR_045147 RNA was transcribed and labeled with biotin RNA (GenePharma). Next, 1 pmol of biotinylated RNA was pretreated with an RNA structure buffer to obtain an appropriate secondary structure. The biotinylated RNA was incubated at 4 °C for 1 hour with 1 mg of the protein extract of PDLSCs, gently mixed with 40 μL of streptavidin beads (Invitrogen), and incubated on a rotator overnight at 4 °C. The beads were washed 5 times with 1× TBST buffer. The proteins were precipitated and diluted in 50 μL of loading buffer at 99 °C for 10 minutes. The collected proteins were detected using Western blotting.

### Development of a critical-size calvarial defect, PDLSC sheet preparation, and implantation

To prepare the PDLSC sheets, MSCs were seeded onto six-well plates at a density of 2 × 10^5^ cells/well. Ascorbic acid (50 M) was added to the culture medium for a week. Subsequently, a PDLSC sheet was formed. A scraper was used to scrape the PDLSC sheet. Nude rats (male, 3-month-old) were used for animal experiments. Anesthesia was administered via intraperitoneal injection containing 4% ketamine/xylazine. Based on previous reports, we created critical-sized defects in the calvaria. Briefly, a sagittal incision was prepared in the calvaria of nude rats, and the skin and periosteal layers were dissected. When the parietal bones were fully exposed, critical-sized defects (5 mm in diameter) were created on both sides of the middle cranial suture by dental implant drills. Subsequently, 2 types of MSC sheets were placed at the defect site: (1) a PDLSCs/Consh sheet and (2) a PDLSCs/NR_045147-sh sheet. The periosteum and skin were overlaid and firmly sutured using an absorbable suture (4/0). Eight weeks after transplantation, the transplanted tissue was harvested.

### Statistical analysis

Each experiment was performed at least thrice. All data were analyzed using SPSS 17 (SPSS Inc.). The significance was determined using the Student’s *t*-test, one-way ANOVA, and the Kruskal-Wallis test. ns: no significant difference. Differences were considered significant at *P* < .05.

## Results

### NR_045147 inhibited osteogenic differentiation and migration of PDLSCs

**Figure 1.**
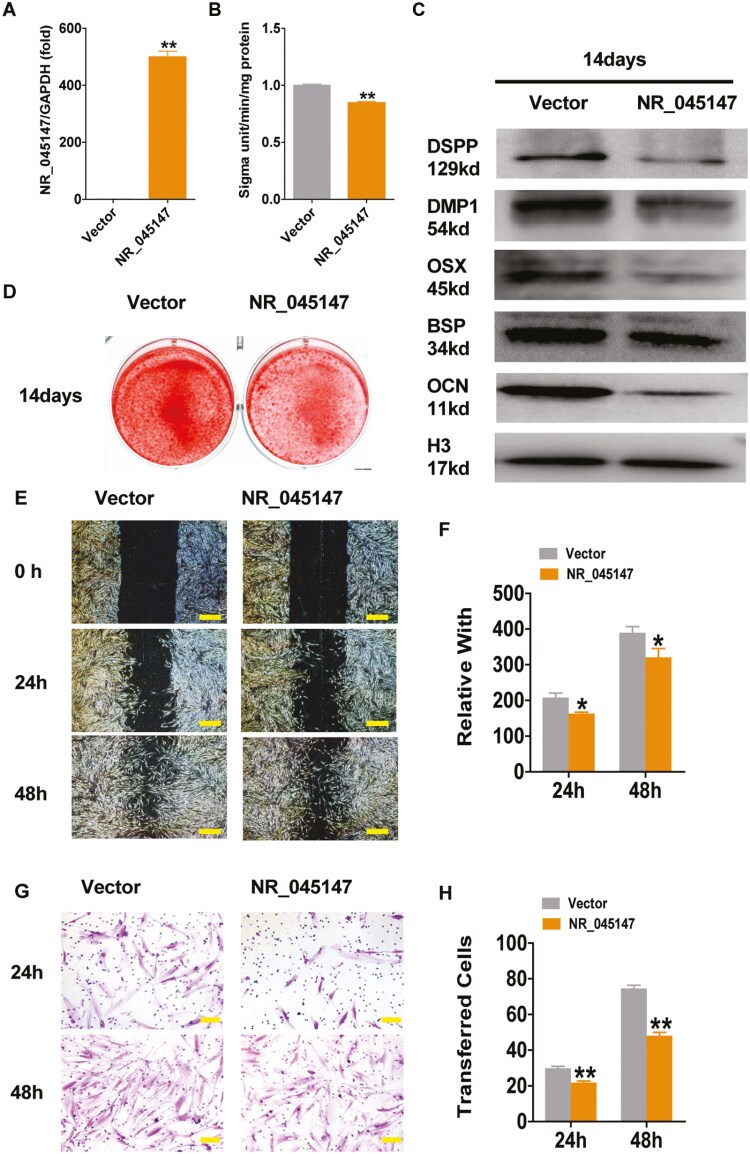
NR_045147 reduced PDLSC osteogenic differentiation and migration potential. (A) qRT-PCR analysis confirming the efficacy of NR_045147 overexpression in PDLSCs. (B) ALP activity was reduced in PDLSCs overexpressing NR_045147. (C) Western blotting revealed lower levels of osteogenesis-related proteins DSPP, DMP1, OSX, BSP and OCN in cells overexpressing NR_045147. (D) Alizarin Red Staining indicating diminished osteogenic calcification in NR_045147-overexpressing cells. (E, F) Scratch assays revealed impaired migration ability in PDLSCs upon NR_045147 overexpression. (G, H) Transwell assays revealed reduced invasion potential in cells with elevated NR_045147 levels. Bar graph: analysis of invading cells. Mean ± SD, (*n* > 3). *P < .05. **P < .01 (*t*-tests).

We analyzed the effects of NR_045147 on bone differentiation and cell migration in vitro. NR_045147-overexpression plasmids were constructed and packaged using lentiviruses and then transfected into PDLSCs. NR_045147 levels were significantly elevated following its overexpression (Figure 1A). An ALP activity assay and alizarin red staining were performed to identify osteogenic changes in PDLSCs. ALP activity and alizarin red staining were detected 3 and 14 days after osteogenesis induction, respectively. Relative to the vector group, the ALP activity was reduced after NR_045147 overexpression (Figure 1B). NR_045147 overexpression resulted in the downregulation of DSPP, DMP1, OSX, BSP, and OCN expression (Figure 1C) and alleviated osteogenic calcification (Figure 1D). After osteogenic differentiation for 14 days, osteogenesis-related factor levels were evaluated using Western blotting. Migration analysis, based on wound healing, revealed that cell migration was significantly inhibited after NR_045147 overexpression (Figure 1E and F). The invasion assay confirmed that NR_045147 overexpression reduced the invasiveness of PDLSC (Figure 1G and H).

To confirm the potential function of NR_045147, we knocked down NR_045147 in PDLSCs via lentiviral transfection. The efficacy of NR_045147 silencing in PDLSCs was evaluated via qRT-PCR (Supplementary Figure 1A). After osteogenic induction for 3 days, NR_045147 knockdown significantly increased ALP activity (Supplementary Figure 1B). Western blotting revealed that NR_045147 knockdown upregulated levels of DSPP, DMP1, OXS, BSP, and OCN proteins relative to those in the control group after 2 weeks of osteogenic induction (Supplementary Figure 1C). Alizarin red staining was performed after 2 weeks of osteogenic induction. NR_045147 knockdown increased calcium mineralization (Supplementary Figure 1D). The wound healing assay confirmed that NR_045147 silencing improved PDLSC migration (Supplementary Figure 1E and F). The Transwell assay revealed that PDLSC invasiveness was significantly greater in NR_045147sh PDLSCs than in Consh PDLSCs (Supplementary Figure 1G and H).

### Knockdown of NR_045147 effectively promoted osteogenesis in vivo

**Figure 2.**
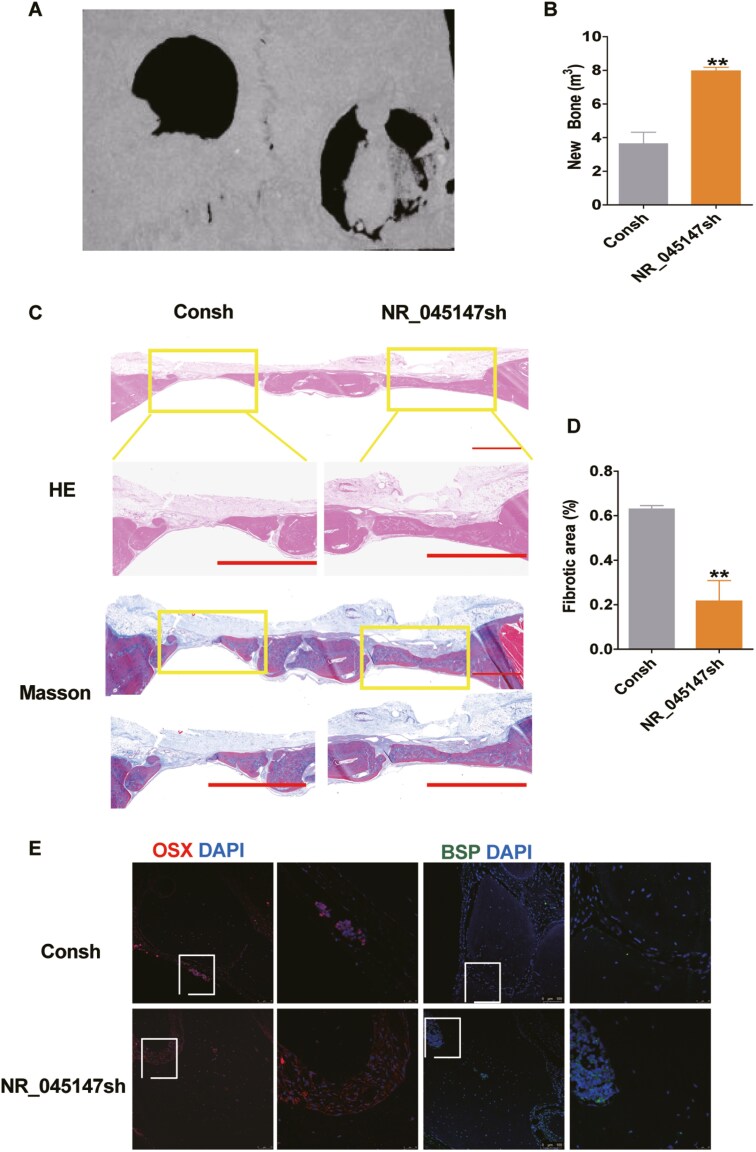
NR_045147-depleted PDLSCs can effectively promote osteogenesis in vivo. (A, B) Implantation of infected cells into cranioparietal bone. Nude mice were divided into 2 groups: Consh and NR_045147sh (*n* = 6). Micro-CT was used to detect bone regeneration at 8 weeks after implantation. (C, D) Histological assessment of new bone formation via H&E and Masson staining. (E) Representative confocal images for OSX and BSP. The nucleus was counterstained with DAPI (blue) (*n* > 6). *P < .05, **P < .01.

To explore the ability of NR_045147 to repair bone defects, we constructed a nude mouse calvaria defect model and used micro-computed tomography to detect bone regeneration 8 weeks after implantation (Figure 2A). The regenerated bone area was larger in the NR_045147sh group than in the Consh group. In addition to the difference in tissue texture, the NR_045147sh group exhibited a more notable expansion of the new bone. Based on the bone volume fraction and mineral density, we quantified the formation of the new bone. The new bone was more clearly discernible at the defect site in the NR_045147sh group than in the control group (Figure 2B). To further elucidate bone defect histomorphology, H&E and Masson staining were performed 8 weeks after implantation. The findings revealed substantial new bone formation, with remarkably better trabecular bone and collagen tissue formation in the NR_045147sh group (Figure 2C and D). The location of the target DNA was directly visualized via fluorescence microscopy, and OSX and BSP were detected in both the cytoplasm and nucleus. The results suggest that OSX and BSP fluorescence was facilitated by NR_045127 inhibition (Figure 2E), presumably because NR_045147 silencing increased in vivo osteogenic potential.

### NR_045147 suppressed mitochondrial respiration in PDLSCs

Mitochondrial respiratory function, represented by the basal/maximal respiration capacity, spare respiratory capacity, and ATP production (revealed via Seahorse XF mitochondrial stress testing), was significantly reduced in PDLSCs after transfection using NR_045147-overexpressing plasmids (Supplementary Figure 2A-E). In contrast, basal respiration was higher in the NR_045147sh group than in the control (Supplementary Figure 2G). Maximum respiratory capacity was enhanced following shRNA lentiviral transfer (Figure 4H). Spare respiratory capacity was significantly elevated (Supplementary Figure 2I), and ATP production was significantly higher in the experimental group than in the control group (Supplementary Figure 2J).

**Figure 3.**
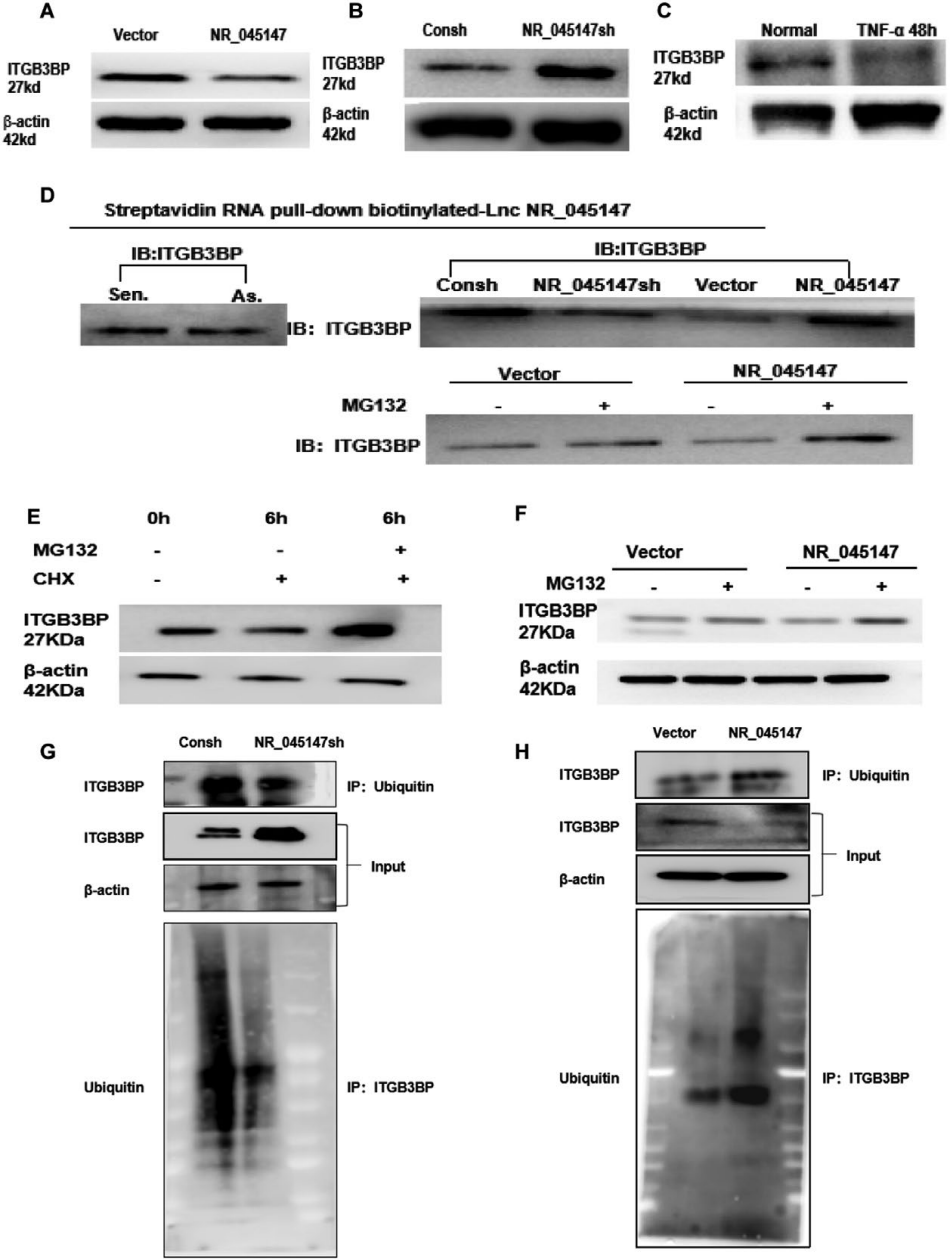
NR_045147 altered ITGB3BP expression in PDLSCs. (A, B) Effects of NR_045147 knockdown or overexpression on ITGB3BP protein stability. (C) ITGB3BP expression was reduced under TNF-α. (D) RNA pull-down assays revealed the binding of NR_045147 to ITGB3BP. (E) Cells were treated with CHX (20 mg/L) for 6 hours before harvest. PDLSC ITGB3BP protein levels were measured. (F) Cells were treated with MG132 (20 mM) for 6 hours before harvest. ITGB3BP levels were measured in NR_045147-overexpressing PDLSCs. (G) NR_045147-knockdown reduced ITGB3BP ubiquitination. (H) NR_045147 overexpression increased ITGB3BP ubiquitination. β-actin was used as a loading control.

**Figure 4.**
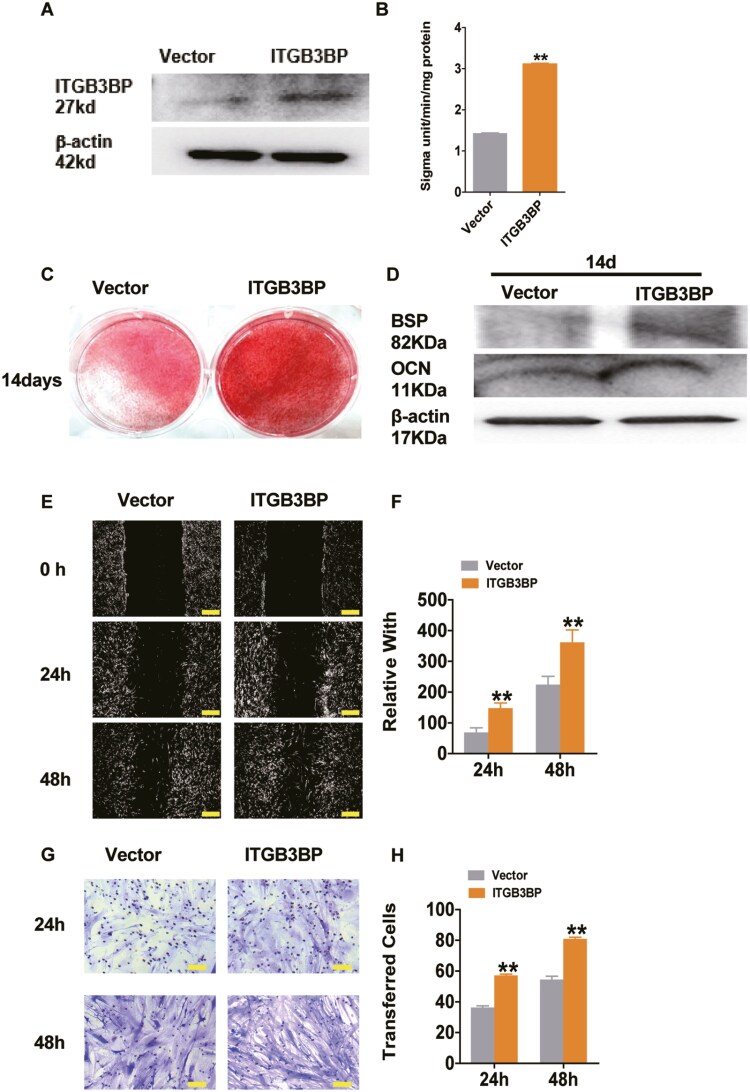
ITGB3BP promoted PDLSC osteogenesis and metastasis. (A) Western blotting confirming ITGB3BP overexpression in PDLSCs. (B) ALP activity was elevated in ITGB3BP-expressing PDLSCs. (C) ARS indicating enhanced PDLSC osteogenic calcification in ITGB3BP-overexpressing cells. (D) Western blotting revealed enhanced levels of osteogenesis-related proteins BSP and OCN in ITGB3BP-overexpressing PDLSCs. (E, F) Scratch assays revealed enhanced migration potential in PDLSCs upon ITGB3BP overexpression. (G, H) Transwell assays revealed enhanced invasion potential in ITGB3BP-overexpressing cells. Bar graph: analysis of invading cells. Mean ± SD (*n* > 3). *P < .05, **P < .01 (*t*-test).

### Loss of NR_045147 rescued osteogenesis under inflammation

To further elucidate the function of NR_045147 during periodontitis-related infection, a culture medium with 10 ng/mL of tumor necrosis factor (TNF)-α was used to recapitulate a model inflammatory environment in vitro. TNF-α significantly increased IL-6 and IL-8 expression in PDLSCs relative to that in the control with no stimulation (Supplementary Figure 3A and B). The expression of *NR_045147*, an important gene involved in immune and inflammatory responses, was upregulated after TNF-α infection (Supplementary Figure 3C). We introduced TNF-α into the culture media of Consh and NR_045147sh cells during osteoblastic differentiation for 14 days. ALP activity and alizarin red staining both revealed the better osteogenic ability of NR_045147sh cells in an inflammatory environment (Supplementary Figure 3D and E). Wound closure was significantly more efficient in the NR_045147sh cells than in the Consh cells; however, this effect was only prominently observed under TNF-α stimulation (Supplementary Figure 3F-G). The same trend was revealed by the results of the transwell assay (Supplementary Figure 3H-I).

### NR_045147 knockdown alleviated TNF-α-mediated inhibitory effect in PDLSCs

We focused on examining the factors affecting the osteogenic differentiation of PDLSCs derived from an inflammatory microenvironment and improving their migratory potential. TNF-α (10 ng/mL) inhibited ALP activity in PDLSCs on day 3 of osteogenic culture; this inhibitory effect was reversed by NR_045147 knockdown (Supplementary Figure 4A). Similarly, TNF-α (10 ng/mL) inhibited osteogenic differentiation of PDLSC. Matrix mineralization by PDLSCs on day 14 was remarkably inhibited by TNF-α; this effect was mitigated after NR_045147 silencing (Supplementary Figure 4B). Following culturing with TNF-α (10 ng/mL), PDLSC cell migration potential was significantly reduced in the Consh group but was elevated (non-significantly) in the NR_045147sh group, relative to Consh group (Supplementary Figure 4C-D). Therefore, NR_045147 silencing enhanced cell viability and inhibited inflammation in TNF-α treated PDLSCs. Thus, NR_045147 can be considered a potential therapeutic target for periodontitis.

### NR_045147 downregulated ITGB3BP expression in PDLSCs

We examined the relationship between *ITGB3BP* and *NR_045147*. NR_045147-overexpressing PDLSCs exhibited reduced *ITGB3BP* levels. *NR_045147* knockdown increased ITGB3BP expression (Figure 3A), whereas NR_045147 overexpression reduced it (Figure 3B). ITGB3BP levels were reduced in PDLSCs following 48 hours of co-culture with TNF-α (Figure 3C). To elucidate the mechanisms underlying the enhancement of PDLSC function by NR_045147, we performed an RNA pull-down assay, followed by screening for NR_045147-associated proteins that might participate in this process. By comparing the NR_045147 precipitate with the antisense and bead control precipitates, ITGB3BP was identified as a potential binding protein for NR_045147. A significant correlation was observed in the NR_045147sh group, whereas the opposite was observed in the NR_045147-overexpressing group (Figure 3D). ITGB3BP degradation in PDLSCs was reduced by treatment with the protein-synthesis inhibitor cycloheximide (CHX) and proteasome-inhibitor MG132 (Figure 3E). NR_045147-induced downregulation of ITGB3BP was blocked in PDLSCs treated with MG132, suggesting that this lncRNA maintains protein stability by competing with ubiquitination (Figure 3F). NR_045147 knockdown reduced ITGB3BP ubiquitination (Figure 3G), whereas its overexpression significantly increased ITGB3BP ubiquitination (Figure 3H). Therefore, NR_045147 may promote proteasome-dependent degradation of ITGB3BP in PDLSCs.

### NR_045147 promoted MDM2-mediated ITGB3BP ubiquitination and degradation

To identify the ubiquitin ligase that might target ITGB3BP for degradation in PDLSCs, we screened for candidate ITGB3BP ligases using BioGRID4.4 (www.thebiogrid.org).^24^ The association between E3 ligases and ITGB3BP was confirmed via co-IP. MDM2 levels were substantially elevated in NR_045147-overexpressing PDLSCs and significantly reduced in NR_045147-depleted PDLSCs (Supplementary Figure 5A and B). Periodontal ligament stem cells co-cultured with TNF-α for 48 hours exhibited elevated MDM2 levels but reduced ITGB3BP levels (Supplementary Figure 5C). The binding between ITGB3BP and MDM2 was significantly reduced by NR_045147 overexpression (Supplementary Figure 5D). Conversely, NR_045147 knockdown enhanced MDM2–ITGB3BP conjugation (Supplementary Figure 5E). Therefore NR_045147 may facilitate ITGB3BP-MDM2 interactions, thus promoting MDM2-induced ITGB3BP ubiquitination and proteasomal degradation.

### ITGB3BP promoted PDLSC osteogenesis and metastasis

We investigated the functional role of ITGB3BP in PDLSCs in vitro. RT-qPCR confirmed lentivirus-mediated ITGB3BP overexpression in PDLSCs (Figure 4A). Alkaline phosphatase activity and alizarin red staining were detected 3 and 14 days after osteogenesis induction, respectively. Based on ALP levels, ITGB3BP-overexpressing cells exhibited better osteogenic ability (Figure 4B). Alizarin red staining and Western blotting revealed that ITGB3BP overexpression significantly promoted PDLSC osteogenic differentiation (Figure 4C and D). Wound scratching and transwell assays revealed that ITGB3BP accelerated migration and enhanced the invasive ability of PDLSCs (Figure 4E-H). Therefore, ITGB3BP promotes osteogenesis and metastasis in PDLSCs.

## Discussion

Periodontitis is an inflammatory disease characterized by the loss of alveolar bone. Animal experimentation has revealed that MSC transplantation promotes bone regeneration and reduces pro-inflammatory cytokine secretion.^25^ In periodontitis, the natural self-repairing potential of alveolar bone is severely compromised owing to the imbalance between pro- and anti-inflammatory macrophages and excessive cytokine excretion, which hampers cellular bioenergetics and resident stem-cell differentiation capacity. The periodontal ligament stem cells exhibit severe mitochondrial dysfunction and reduced ATP production.^26^ While PDLSCs possess osteogenic differentiation potential and can be used for alveolar bone regeneration,^27^ their osteogenic differentiation is inhibited in an inflammatory microenvironment.^28,29^ Cell migration is central to many biological processes, including osteogenic differentiation, and mitigation of infection and bone defects.^30^ Inflammatory cytokines, such as TNF-α, inhibit fibrosis and fibroblast migration in scar tissue.^31^ Upregulation of mitochondrial metabolism is a feasible technique for enhancing osteogenic function in vitro and promoting bone defect repair in situ.^32^ Presumably, as periodontitis worsens, inflammatory factors mediate an increase in NR_045147 levels.

This study confirmed that NR_045147 depletion significantly improved PDLSC osteogenesis and metastasis, suggesting its importance in modulating MSC differentiation and development. The in vivo data verified that NR_045147 depletion enhanced bone regeneration in PDLSCs. NR_045147 overexpression reduced mitochondrial respiration. NR_045147 expression was elevated in PDLSCs under inflammatory conditions. TNF-α reduced PDLSC osteogenic and migration ability. Reduction of NR_045147 levels may alleviate the negative effects of inflammation on tissue regeneration. Therefore, NR_045147 may participate in multiple biological functions, with pivotal regulatory roles in periodontitis.

The inflammatory cytokine network participates in periodontitis and affects osteoclastic activity and alveolar bone loss. Both the IL-6 family and TNF have pleiotropic effects on lymphocyte promotion and tissue destruction and are recognized as pro-inflammatory cytokines.^33^ IL-8 and its polymorphisms are associated with an increased risk of periodontal disease.^34^ The presence of bacterial metabolic products and other substances (lipopolysaccharides, enzymes, and toxins) enhances proinflammatory cytokine expression and the release of active agents, promoting local tissue-lesion development.^35^

*ITG3BP*, a chromatin remodeling-related gene, is downregulated during the immune response. ITGB3BP is closely associated with cancer, but its role in MSCs is unclear.^36,37^ Here, pre-ITGB3BP depicted 5 isoforms, with splicing at its 5ʹ alternative splice site generating mature NR_045147. This transcript variant lacks an alternative in-frame exon in the 5ʹ coding region, and its primary open reading frame has a stop codon located >50 nt from its terminal splice site (www.ncbi.nlm.nih.gov). Similar transcript-variant models of mRNA and lncRNA, such as that of *Sox2* and its overlapping transcript, have been reported.^38,39^ Various types of lncRNAs exhibiting alternative splicing approaches, and their potential clinical therapeutic value, have been reported.^40^ However, the precise mechanisms underlying lncRNA biogenesis and processing remain unclear. Therefore, the biogenesis of the NR_045147 requires further investigation. Some lncRNAs that are structurally similar to mRNAs exhibit functional uniqueness by participating in and modulating various cellular processes, such as histone modification, DNA methylation, and transcription.^41^ Different alterations in transcription result in better-targeted therapies with fewer side effects.^42^

Integrins are cell-surface adhesion receptors that play important roles in mediating many physiological processes, including inflammation, cell motility, osteogenic differentiation, and mitochondrial metabolism.^43,44^ ITGB3BP is the focus of current research because of its association with integrins. Integrin beta3-binding protein has been identified as a therapeutic target in cancer treatment, significantly influencing cancer cell metastasis and invasion.^45^ ITGB3BP may participate in pathological conditions beyond cancer, including cardiovascular diseases and inflammatory disorders.^46^ Under hypoxia, ITGB3BP reduces nuclear factor kappa B (NFκB) activation and NFκB binding to the HIF-1α promoter, subsequently preventing hypoxic endothelial proliferation and angiogenic responses.^47^ ITGB3BP may regulate kinetochore-microtubule attachment stability and ensure accurate chromosomal segregation during mitosis.^48^ Integrin beta3-binding protein participates in cell-cycle regulation and proteostasis.^49^ ABCB-1 inhibits both mitochondrial transport along microtubules and BMP signaling by downregulating ITGB3BP.^50^ The current research on ITGB3BP underscores its significance in cellular physiology and disease pathogenesis, offering promising avenues for future therapeutic interventions and diagnostic advancements.

Here, NR_045147 negatively downregulated ITGB3BP. Integrin beta3-binding protein expression was significantly reduced in NR_045147-overexpressing cells and enhanced in NR_045147-silenced cells. Using in vitro transcribed biotinylated NR_045147 to identify NR_045147-interacting proteins, the RNA pull-down assay revealed enrichment of ITGB3BP. Integrin beta3-binding protein is an essential mediator of the interaction between ITGB3 and other cellular components, thus contributing substantially to cellular communication and adhesion.^51^ Via its molecular interactions, ITGB3BP regulates cell migration, proliferation, and differentiation via molecular interactions, thereby influencing various physiological and pathological processes. By modulating the function of ITGB3, ITGB3BP affects crucial cellular activities, including wound healing, tissue regeneration, and immune responses.^52,53^ ITGB3BP participates bilaterally in cancer development, functioning as a tumor suppressor or promoter.^54^ Hence, it is central to the cell adhesion network and signaling pathways, critically mediating cellular functions essential for development, homeostasis, and disease progression.

Ubiquitination, a key post-translational modification, is central to maintaining cellular protein homeostasis.^55^ Ubiquitin is a small regulatory protein that binds to specific substrates for proteasomal degradation, regulating transcription and translation, cell homeostasis and proliferation, DNA repair, and signal activation and suppression.^56,57^ The ubiquitin-proteasome system regulates protein synthesis by influencing RNA metabolism.^58^ Non-coding RNAs (ncRNAs) participate extensively in mitophagy via ubiquitin.^59^ ncRNAs regulate E3 ubiquitin ligase levels and target protein ubiquitination-induced degradation, and vice versa. A few E3 ubiquitin ligases regulate ncRNA expression.^60^ Ubiquitination represents a new strategy for developing effective ncRNA therapies. Our results highlight this potential: NR_045147 modulated ITGB3BP ubiquitination, suggesting a sophisticated regulatory role in proteasomal degradation pathways. NR_045147 promoted ITGB3BP binding to its E3 ubiquitin ligase, potentially increasing its susceptibility to ubiquitination and subsequent proteasomal degradation, as reflected by the augmented ubiquitin signal under NR_045147-knockdown conditions. The ITGB3BP ubiquitination profile responded markedly to changes in NR_045147 levels, indicating that ncRNA may directly influence the ubiquitin-proteasome system. The MG132-induced protective effect against proteasomal degradation supports the hypothesis that ITGB3BP degradation is ubiquitin-mediated, and NR_045147 plays a pivotal role in this regulatory pathway. Crucially, the function of MDM2 as an E3 ubiquitin ligase was highlighted by its involvement in ITGB3BP ubiquitination. Inflammatory conditions, such as those induced by TNF-α, augment NR_045147 expression, which can, in turn, enhance MDM2 activity, increasing ITGB3BP ubiquitination and degradation. This elucidates the substantial role of MDM2 in the post-translational modification landscape under the influence of NR_045147 and underscores the potential of targeting these molecular interactions in developing new therapeutic strategies for diseases in which protein homeostasis is disrupted.

Proteolysis results in the formation of beta3-endonexin (EN-L). While the presence of a calpain inhibitor prevents the formation of EN-L, adding calpain to platelet lysates induces its formation.^61^ Here, NR_045147 promoted the ITGB3BP-MDM2 interaction, enhancing ITGB3BP ubiquitination and degradation. MDM2, an E3 ubiquitin ligase that negatively regulates p53 transcription,^62^ stem cell differentiation,^63^ mitochondrial respiration,^64^ and T-cell immunity^65^ and participates in diverse cellular functions. It interacts with mitochondrial proteins, substantially altering mitochondrial function. It binds to mitochondrial membrane proteins, governing membrane permeability and stability, thereby affecting mitophagy.^66^ It negatively regulates NADH, thus reducing mitochondrial respiration and influencing mitochondrial oxidative phosphorylation and ATP synthesis.^67^ By restoring mitochondrial membrane potential, MDM2 may participate in mitochondrial DNA replication and repair.^68^ These interactions underscore the importance of ITGB3BP and MDM2 in modulating mitochondrial function and cellular physiology. Further exploration of the interactions between ITGB3BP, MDM2, and mitochondrial proteins will provide valuable insights into cellular homeostasis and disease pathogenesis.

Mitochondria provide cellular energy and participate centrally in osteogenic differentiation, migration, and intracellular environmental homeostasis. During osteogenic induction, the dynamics and functions of local mitochondria are altered. Mitochondrial regulation affects osteoblast and osteoclast activity, influences its direction, and determines the final identity of the differentiated cells.^69,70^ Mitochondria are essential in maintaining intracellular Ca^2+^ homeostasis.^71,72^ They regulate osteoblast function by influencing intracellular signaling pathways, such as the Wnt-β-catenin signaling pathway, which is vital for bone formation.^73^ Mitochondria generate ATP via oxidative phosphorylation, providing the energy necessary for cytoskeletal rearrangement and for the signaling pathways that are essential for cell migration-related processes such as adhesion, protrusion formation, and directional sensing.^74^ Mitochondrial dysfunction, characterized by impaired oxidative phosphorylation and increased production of metabolic products, can exacerbate inflammation by activating stress-responsive kinases and transcription factors involved in inflammatory gene expression.^75^ Here, NR_045147 significantly altered mitochondrial respiration in PDLSCs. NR_045147 overexpression markedly reduced both basal and maximal mitochondrial respiration, suggesting that NR_045147 may compromise the capacity of mitochondria to produce ATP. This effect may be directly related to the diminished osteogenic differentiation and migratory capability observed here in PDLSCs. NR_045147 overexpression reduced the ability of PDLSCs to adapt to metabolic stress, potentially contributing to their suppressed regenerative potential under inflammatory conditions.

## Conclusions

The impact of NR_045147 on PDLSC osteogenic differentiation, migration, and mitochondrial metabolism was examined. Reducing NR_045147 expression increased ITGB3BP levels by reducing its ubiquitination by MDM2, in turn promoting PDLSC osteogenic differentiation, migration, and mitochondrial respiration. These findings highlight the potential of NR_045147 as a therapeutic target for improving periodontal regeneration and treatment outcomes.

## Supplementary material

Supplementary material is available at *Stem Cells Translational Medicine* online.

szae088_suppl_Supplementary_Tables_1_Figures_1-5

## Data Availability

The data used to support the findings of this study are included within the article.
