# Supplementary material for: LncRNA NR_045147 modulates osteogenic differentiation and migration in PDLSCs via ITGB3BP degradation and mitochondrial dysfunction
Source: Stem Cells Transl Med. 2024 Dec 14;14(2):szae088. doi: 10.1093/stcltm/szae088 (PMC11878762; doi:10.1093/stcltm/szae088)
Supplement: szae088_suppl_Supplementary_Tables_1_Figures_1-5 [file szae088_suppl_supplementary_tables_1_figures_1-5.pdf]

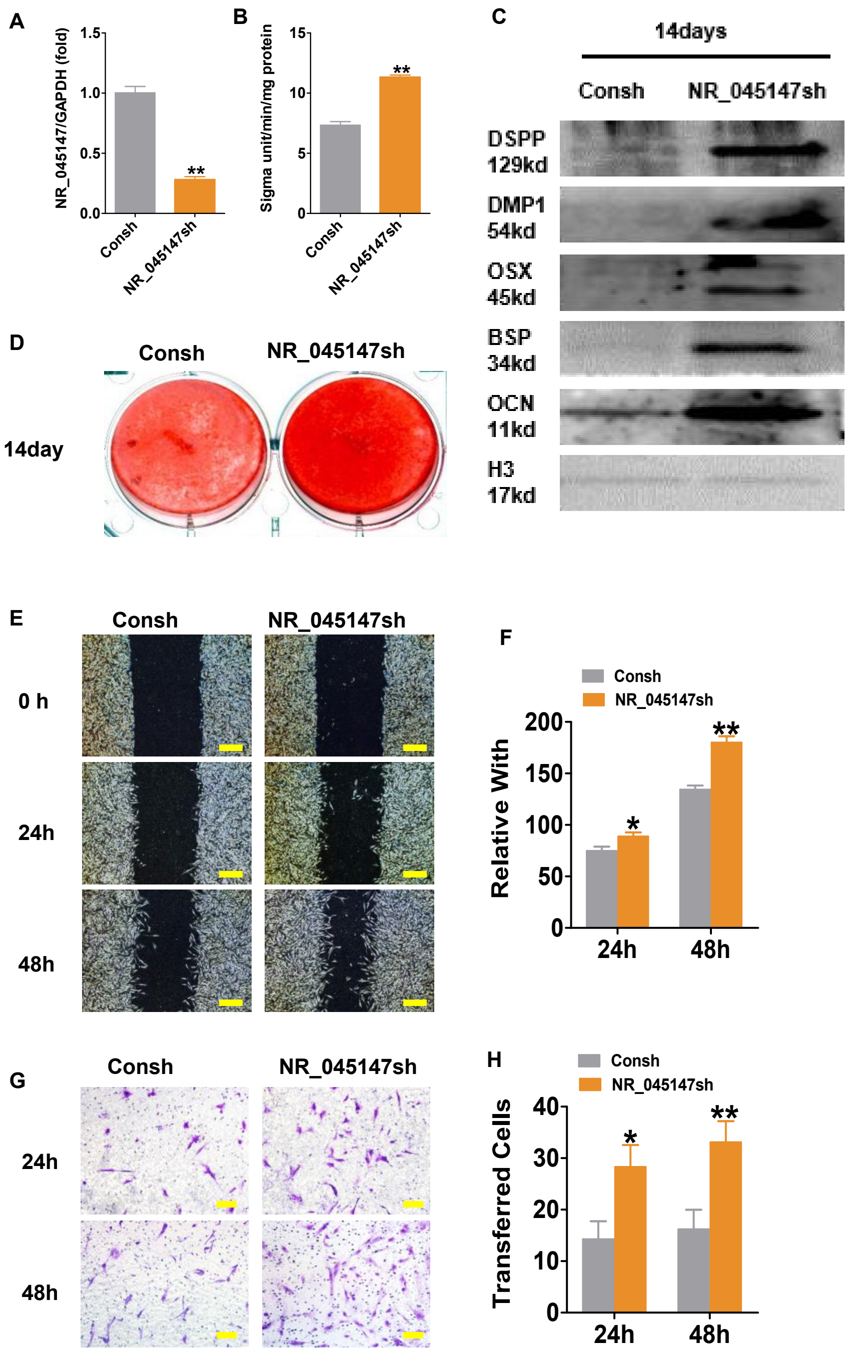

Supplemental Figure 1. NR_045147 knockdown increased PDLSC osteogenic differentiation and migration potential. (A) qRT-PCR analysis confirming the efficacy of NR_045147-knockdown in PDLSCs. (B) ALP activity was enhanced in PDLSCs after NR_045147 knockdown. (C) Western blotting revealed higher levels of osteogenesis-related proteins DSPP, DMP1, OSX, BSP and OCN in cells following NR_045147 silencing. (D) ARS indicating diminished osteogenic calcification in NR_045147-silenced cells. (E, F) Scratch assays revealing recovery of PDLSC migration potential upon NR_045147 silencing. (G, H) Transwell assays revealing increased invasion potential in cells with reduced levels of NR_045147. Bar graph: analysis of invading cells. Mean ± SD (n > 3). *p < 0.05. **p < 0.01 (t-tests).

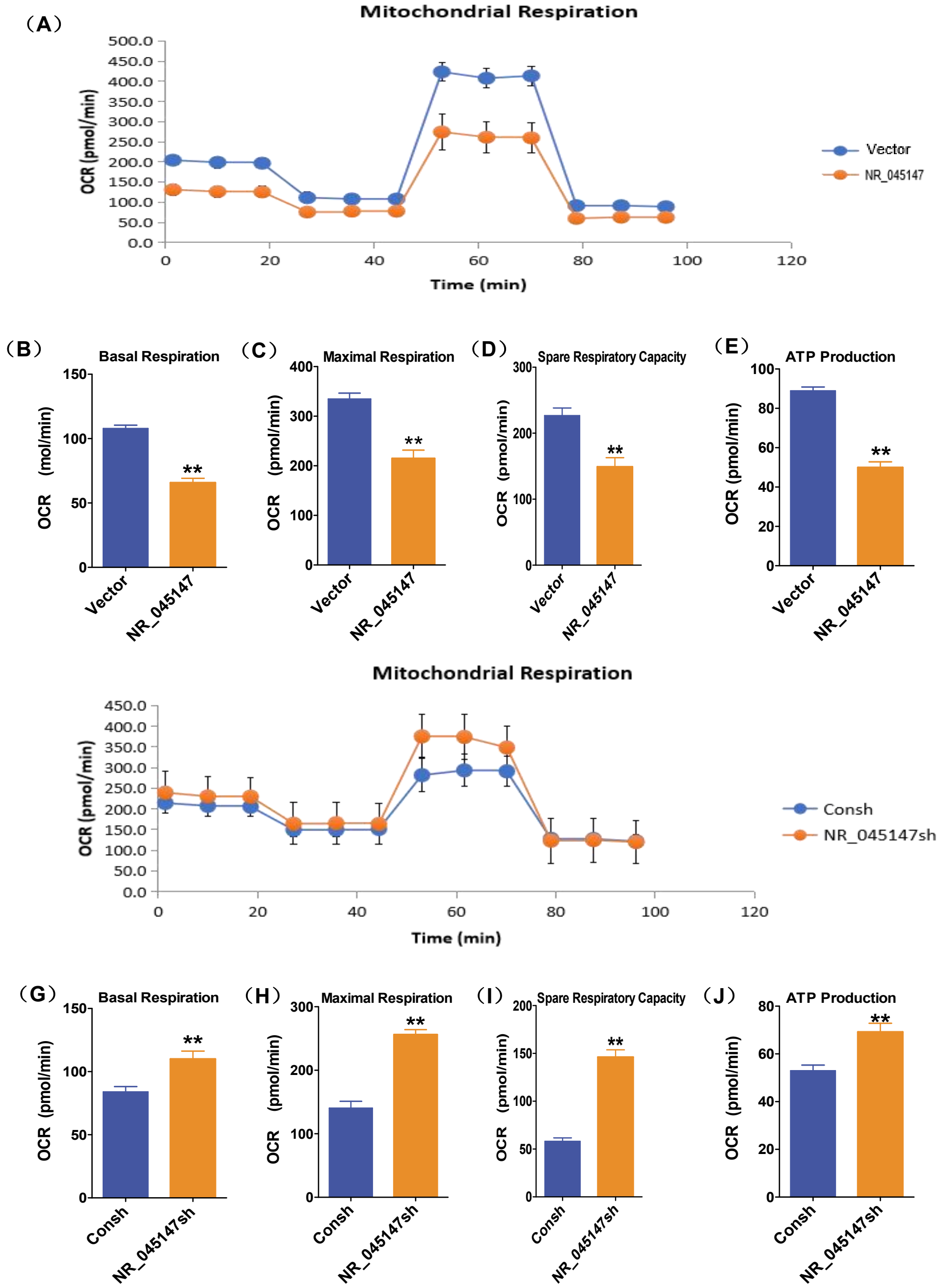

Supplemental Figure 2. NR_045147 inhibited PDLSC mitochondrial respiration. (B, G) Basal respiration analysis confirming NR_045147-induced suppression of the O_2 consumption rate (OCR) in PDLSCs. (C, H) Maximal respiration was enhanced in PDLSCs after NR_045147 knockdown. (D, I) Spare respiratory capacity was diminished in LncRNA NR_045147-overexpressing cells. (E, J) ATP production was elevated in NR_045147-silenced cells. Bar graph: analysis of invading cells. Mean ±SD, (n > 3) *$p < 0.05$. **$p < 0.01$. $p < 0.05$. **$p < 0.01$ (*t*-tests).

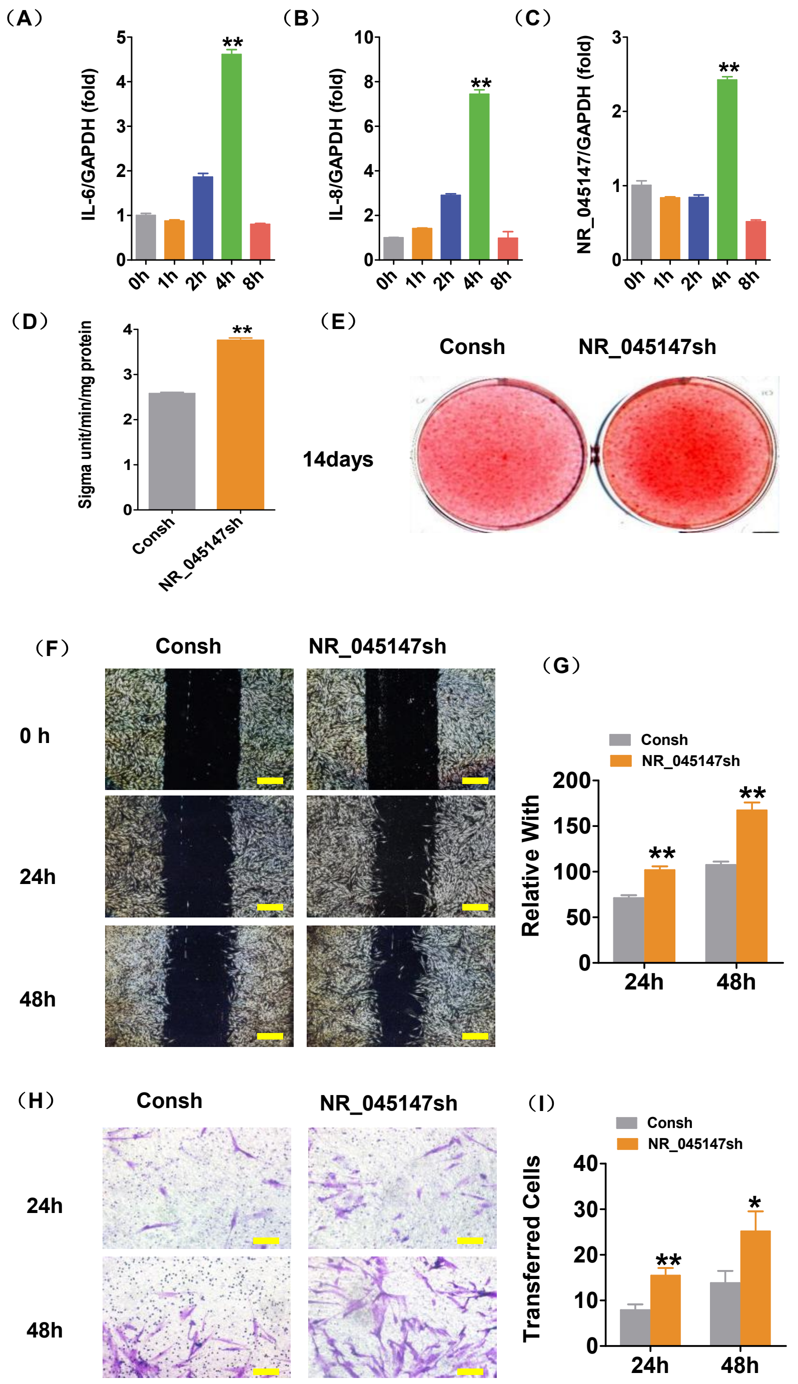

Supplemental Figure 3. Loss of NR_045147 rescued osteogenesis under inflammation. TNF-α promoted inflammatory cytokine and NR_045147 production. (A-C) mRNA expression of (A) IL-6, (B) IL-8, and (C) NR_045147. (D) ALP activity, measured using a test kit. (E) Osteogenic calcification (Alizarin Red staining). (F-G) Migration potential (the scratch method). (H-I) Invasion ability (Transwell assay). Mean ± SD (n > 3). * $p < 0.05$, ** $p \leq 0.01$ (*t*-tests).

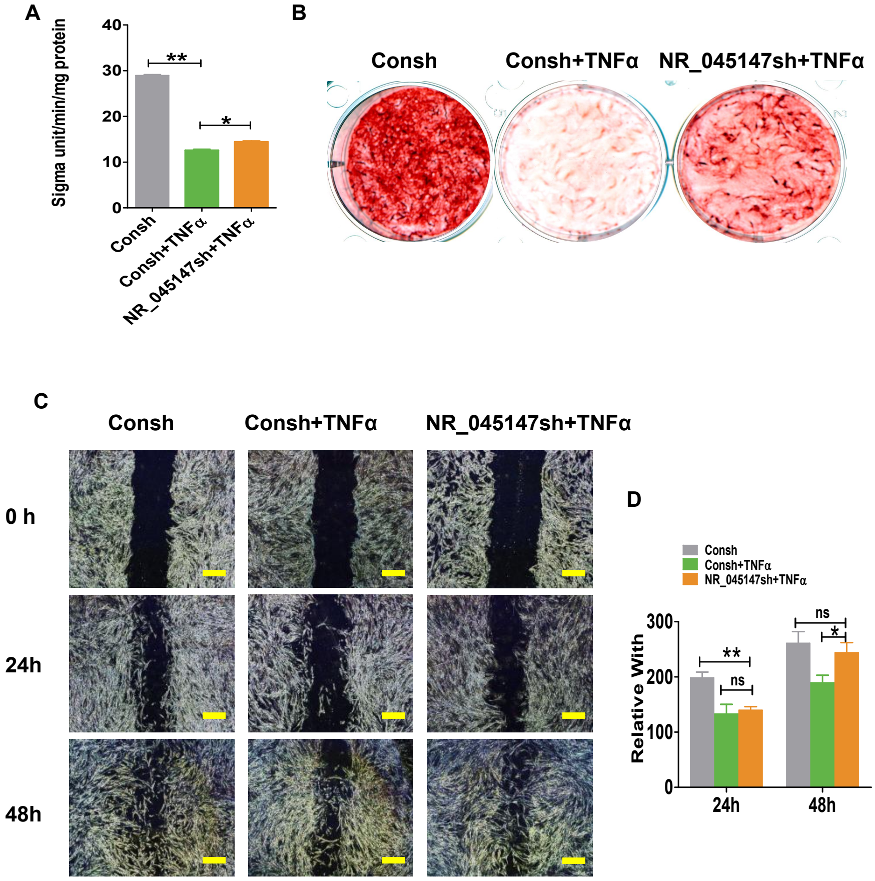

Supplemental Figure 4. NR_045147-knockdown rescues TNF-α-inhibited osteogenic differentiation and migration of PDLSCs. (A) PDLSC ALP activity was enhanced by NR_045147 knockdown under TNF-α. (B) PDLSC osteogenic calcification was elevated following NR_045147 knockdown under TNF-α. (C-D) PDLSC migration potential was rescued by NR_045147-silencing under TNF-α. Mean±SD (n > 3) *$p < 0.05$. **$p < 0.01$ (*t*-tests).

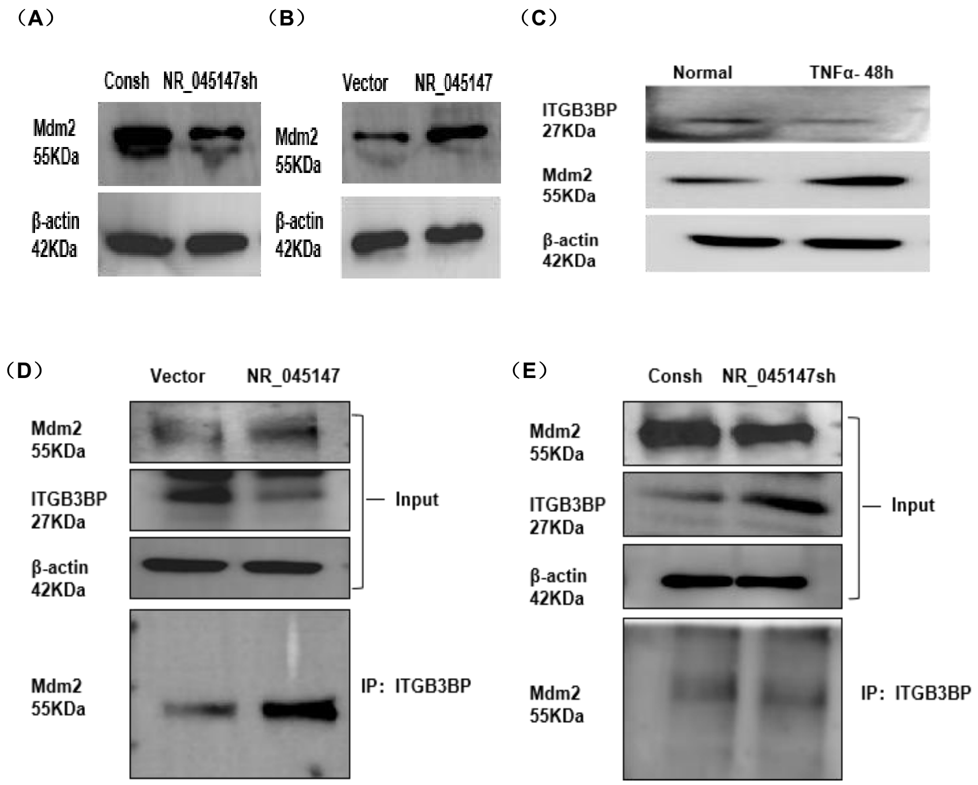

Supplemental Figure 5. NR_045147 promoted MDM2-mediated ITGB3BP ubiquitination and degradation. (A, B) Western blotting of MDM2 in PDLSCs with NR_045147 knockdown (via NR_045147sh) or overexpression, relative to the Consh and Vector groups. (C) Comparison of ITGB3BP and MDM2 levels in PDLSCs under normal conditions and after 48 h of TNF-α stimulation. (D) IP analysis showing the interaction of MDM2 with ITGB3BP in NR_045147-overexpressing PDLSCs versus the vector control. (E) IP analysis demonstrating the association between MDM2 and ITGB3BP in NR_045147-knockdown PDLSCs relative to the control (Consh). β-actin was used as a loading control.
